# *S*-allylmercaptoglutathione Is a Substrate for Glutathione Reductase (E.C. 1.8.1.7) from Yeast (*Saccharomyces cerevisiae*)

**DOI:** 10.3390/antiox7070086

**Published:** 2018-07-06

**Authors:** Tobias Horn, Wolfgang Bettray, Alan J. Slusarenko, Martin C. H. Gruhlke

**Affiliations:** 1 Department of Plant Physiology, RWTH Aachen University, 52056 Aachen, Germany; tobias.horn@rwth-aachen.de (T.H.); alan.slusarenko@bio3.rwth-aachen.de (A.J.S.); 2 Institute of Organic Chemistry, RWTH Aachen University, 52056 Aachen, Germany; Bettray@rwth-aachen.de

**Keywords:** allicin, thiosulfinate, garlic, glutathione reductase

## Abstract

Allicin (diallylthiosulfinate) is a potent thiol reagent and natural defense substance produced by garlic (*Allium sativum*) tissues when damaged. Allicin acts as a redox toxin and oxidizes the cellular glutathione (GSH) pool producing *S*-allylmercaptoglutathione (GSSA). The cellular enzyme glutathione reductase (GR) uses NADPH to reduce glutathione disulfide (GSSG) back to GSH and replenishes the GSH pool. It was not known whether GR could accept GSSA as a substrate. Here, we report that GR from yeast (*Saccharomyces cerevisiae*) shows Michaelis–Menten kinetics with GSSA as substrate in vitro (*K*_m_ = 0.50 mM), but that GSSA is not as good a substrate as GSSG (*K*_m_ = 0.07 mM). Furthermore, cells unable to synthesize GSH because the γ-glutamylcysteine synthetase (*GSH1*) gene is deleted, cannot grow without GSH supplementation and we show that the auxotrophic requirement for GSH in *Δgsh1* mutants can be met by GSSA in the growth medium, suggesting that GSSA can be reduced to GSH in vivo.

## 1. Introduction

Glutathione (GSH) is the major low molecular weight thiol in most eukaryotic and many prokaryotic cells and is regarded as one of the cell’s first lines of defense against several oxidative insults [1,2]. In healthy non-stressed cells, usually nearly all of the GSH in the cytosolic glutathione pool is in the reduced form, reflecting the highly reducing environment of that cell compartment. Indeed, recent estimates using reduction-potential-dependent fluorescent protein probes suggest a cytosolic *E*_GSH_ of <−320 mV in yeast. Assuming a cytosolic [GSH] of 10 mM would mean a ratio of GSH:GSSG of 50,000:1 [3].

**Scheme 1:**
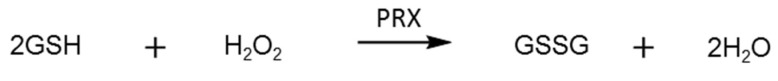
Two mol reduced glutathione (GSH) react with one mol hydrogen peroxide to yield one mol glutathione disulfide (GSSG) and two mol water. In cells the reaction is catalyzed by peroxiredoxin enzymes (PRX).

**Scheme 2:**
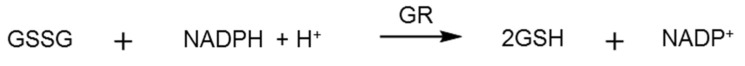
One mol glutathione disulfide (GSSG) is reduced to 2 mol GSH by one mol NADP^+^ + H^+^ under catalysis by glutathione reductase (GR).

The dedicated biosynthetic pathway to GSH consists of two steps. The enzyme γ-glutamylcysteine synthetase (GSH1) catalyzes the coupling of glutamate with cysteine and the γ-glutamylcysteine product is coupled with glycine by glutathione synthetase (GSH2) to make the final product. Cells deleted for the *GSH1* gene must be supplemented with GSH for survival and growth. Upon oxidative stress, caused for example by reactive oxygen species, GSH is oxidized to its dimeric form glutathione disulfide GSSG (Scheme 1). The reaction with H_2_O_2_ is rather slow and in vivo is catalysed by enzymes called peroxiredoxins [4,5]. Furthermore, under oxidative stress conditions, cysteine thiols in proteins may be reversibly glutathiolated to protect them from over-oxidation to sulfenic and sulfinic acid residues [6]. The GSH pool within the cell is restored by the reduction of GSSG to GSH by the NADPH-dependent enzyme glutathione reductase (GR, E.C. 1.8.17) (Scheme 2) [1] whereas glutathiolated proteins are usually reduced by glutaredoxins [7].

**Scheme 3:**
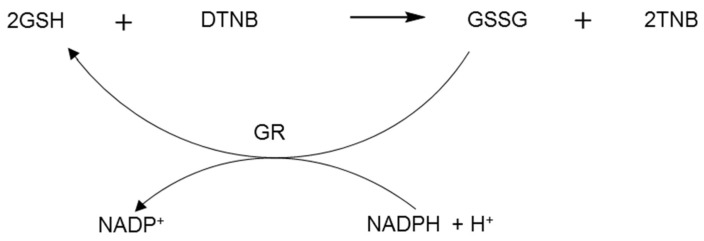
Basis of the GR enzyme assay [8]. GSH is oxidized to GSSG by DTNB and reduced back to GSH by GR. The rate of TNB production is proportional to the concentration of GSH and can followed spectrophotometrically. DTNB: (5,5′-dithiobis-(2-nitrobenzoic acid).

A GR/NADPH-dependent cycling assay was developed to determine [GSH] and [GSSG], whereby GSH is oxidized to GSSG by 5,5′-dithiobis-2-nitrobenzoate (DTNB) in the presence of excess enzyme (Scheme 3) [8]. The 2-nitro-5-thiobenzoate (TNB) formed can be measured spectrophotometrically (λ_max_ = 412 nm) and its rate of production depends upon the rate of GSH formation due to GR activity with GSSG as substrate. In contrast, under conditions of substrate excess, the rate of TNB production is limited by the amount of GR activity present and the assay can be used to measure GR activity.

**Scheme 4:**
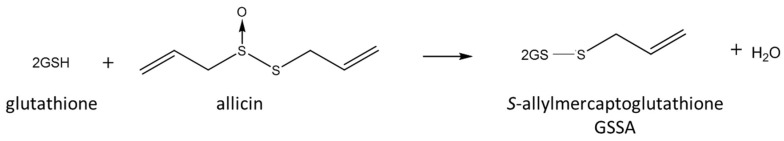
Two mol reduced glutathione (GSH) react with one mol allicin to yield two mol *S*-allylmercaptoglutathione.

Allicin (diallylthiosulfinate) is produced in garlic when cells are damaged and the enzyme alliinase mixes with its substrate alliin which is separately compartmentalized in the cell. A single clove of garlic can produce up to 5 mg of allicin [9], which is the first and major volatile sulfur compound produced and gives fresh garlic its characteristic odor. Allicin was identified as the major antimicrobial substance produced by garlic [10,11] and it was shown to oxidize and deplete the cellular GSH pool, reacting with GSH to yield *S*-allylmercaptoglutathione (GSSA) (Scheme 4). The thiosulfinate group in allicin reacts readily with thiols, particularly in the thiolate ion form, without the need for enzymic catalysis [12,13,14]. Furthermore, allicin can react with accessible cysteine thiols in proteins by *S*-thioallylation [6,15].

**Scheme 5:**
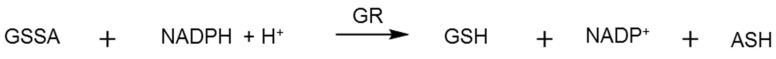
Theoretical NADPH + H^+^-dependent GR-catalysed reduction of one mol GSSA would yield one mol reduced glutathione (GSH) and one mol allylmercaptan (ASH).

GSH and GR have been shown to play an important role in the resistance of cells against allicin [16,17]. GR replenishes the GSH pool from GSSG and this protective effect would be strengthened if GSSA were also a substrate for GR. In this case one mol GSSA would be reduced to one mol each of GSH and allylmercaptan (ASH) (Scheme 5). The goal of this investigation was to clarify whether GSSA can serve as a substrate for GR and commercially available GR from yeast was chosen for this purpose. However, the DTNB assay is not suitable to assay for GR activity with GSSA as a substrate because any GSH formed by GR action on GSSA would be oxidized to GSSG by the DTNB and this would enter into the cycling reaction and lead to complicated mixed-substrate kinetics. Therefore, in order to test whether GSSA is a substrate for GR it is necessary to leave out the DTNB reagent and measure the disappearance of NADPH + H^+^, which can be observed as a reduction in A_340_ over time. In the work reported here we show that indeed GSSA is a substrate for GR from yeast, but has a higher *K*_m_ and therefore a lower substrate affinity with the enzyme than GSSG.

## 2. Materials & Methods

### 2.1. Allicin Synthesis

Allicin was synthesized as previously described by the formic acid-catalyzed oxidation of redistilled diallyl disulfide by hydrogen peroxide [18].

### 2.2. Synthesis and LC-MS of S-allylmercaptoglutathione

S-allylmercaptoglutathione was synthesized after a method modified from Miron et al. [12]. GSH (400 mg dissolved in 5 mL distilled H_2_O) were added dropwise with stirring to 130 mg allicin dissolved in 2 mL 50% (*v*/*v*) methanol and stirred for a further 2 h at room temperature. The white precipitate (yield 450 mg) was washed repeatedly with dichloromethane and the product gave a single peak which eluted from HPLC at 5.8 min (conditions as in [18]).

The identity of the product was confirmed by electrospray ionization mass spectrometry (ESI-MS). Measurements were carried out on a Thermo Fisher Scientific Orbitrap XL (in high resolution mode using methanol/water (50%/50%) with 0.1 mM acetic acid.

### 2.3. Glutathione Reductase Assay

Yeast glutathione reductase was purchased from Sigma-Aldrich GmbH, Steinheim, Germany. The GR enzyme solution (20 U mL^−1^) was prepared in 143 mM sodium phosphate buffer (pH 7.5) containing 6.3 mM Na_2_EDTA. DTNB (5,5’-dithiobis-(2-nitrobenzoic acid), 6 mM) and NADPH (0.3 mM) were prepared in phosphate buffer. The assay mix for GSSG with DTNB present was as follows: 12.5 µL substrate solution (1.0, 0.5, 0.25, 0.1 mM), 5 µL GR solution, 50 µL DTNB solution, 350 µL NADPH solution and 332.5 µL H_2_O. The assay mix for GSSA without DTNB present was as follows: 12.5 µL substrate solution (7.5, 5.0, 4.5, 3.0 mM), 5 µL GR solution, 350 µL NADPH solution and 382.5 µL H_2_O. Substrate concentration ranges giving linear kinetics were established at enzyme excess ensuring the rate of substrate conversion was proportional to the substrate concentration.

### 2.4. Yeast

The haploid *Saccharomyces cerevisiae* yeast strain BY4742 (Matα; his3∆1; leu2∆0, lys2∆0, ura3∆0) was used. The BY4742 mutant ∆*gsh1* (Y17097) used in this study lacks gene for γ-glutamylcysteine synthetase (YJL101C) which catalyzes the first step in glutathione biosynthesis. The mutant was obtained from the EUROSCARF Collection, University of Frankfurt (Main), Germany (http://www.euroscarf.de/).

Yeast was grown in complete synthetic mixture (CSM) medium (0.79 g L^−1^ CSM Drop-Out: Complete [ForMedium, Norwich, United Kingdom]; 6.9 g L^−1^ Yeast Nitrogen Base [ForMedium, Norwich, United Kingdom]; 40 g L^−1^ D-Glucose [Carl Roth, Karlsruhe, Germany], 15 g L^−1^ agar for solid medium.

## 3. Results and Discussion

### 3.1. Synthesis of GSSA

**Figure 1:**
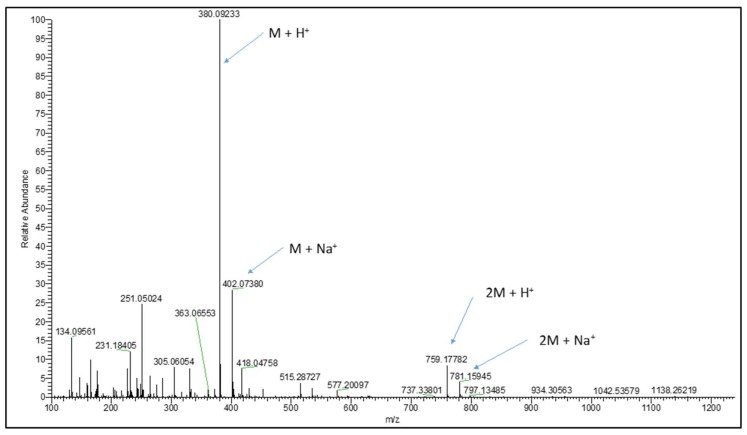
LC-MS (ESI) of *S*-allylmercaptoglutathione (GSSA) synthesized by reacting glutathione (GSH) with allicin.

Pure GSSA was needed for the enzyme assay and was prepared as described in the Methods section. The product of the synthesis gave a single HPLC peak eluting at 5.8 min and the identity was determined by LC-MS using ESI-Ionization (Figure 1). The major peak at *m*/*z* 380.1 corresponds to GSSA + H^+^ (labelled in the Figure 1 as M + H^+^) with a further peak at 402.1 corresponding to GSSA + Na^+^ and minor peaks for the corresponding dimers at 759.2 and 781.2, respectively (Figure 1).

### 3.2. Glutathione Reductase Assays

**Figure 2:**
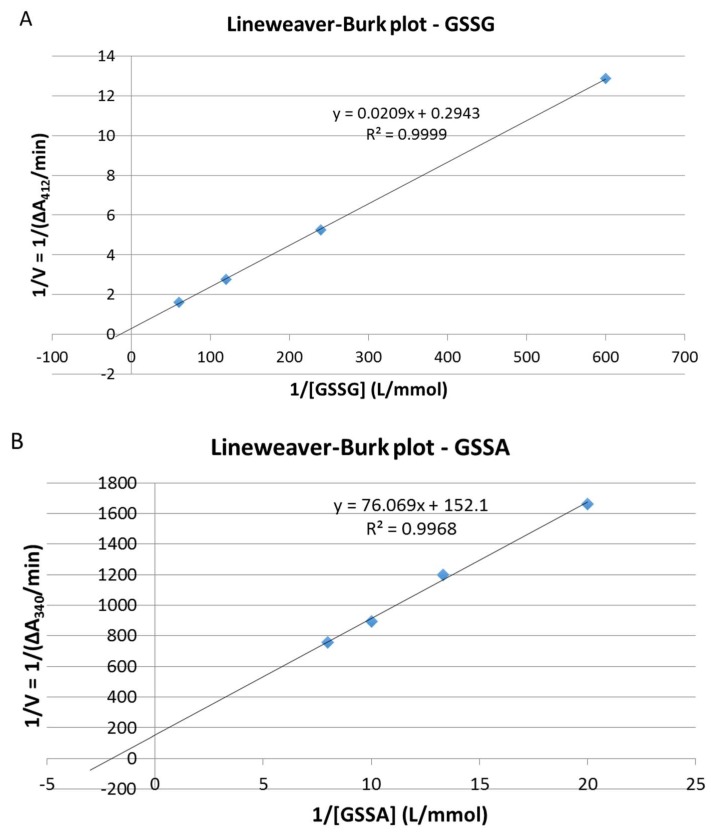
(**A**) Lineweaver-Burk plot for glutathione reductase (GR) with glutathione disulfide (GSSG) + 5,5’-dithiobis-2-nitrobenzoate (DTNB). (**B**) Lineweaver-Burk plot for GR with GSSA without DTNB. The point at which the line intersects the x axis = −1/*K*_m_. The data from which the individual points were calculated are shown in Supplementary Figure S1A,B and Supplementary Tables S1 and S2.

Glutathione reductase was purchased from Sigma-Aldrich and used in assays with GSSG and GSSA as substrates with and without DTNB, respectively. The reaction showed first order kinetics with both substrates and suitable substrate concentrations under conditions of excess enzyme were determined empirically. Lineweaver-Burk plots prepared for GSSG and GSSA as substrates are shown in Figure 2A,B. The point at which the line intersects the x axis = −1/*K*_m_ and the respective *K*_m_ values for these substrates with GR, 0.07 mM for GSSG and 0.50 mM for GSSA, were calculated from the slopes of the respective lines.

The Michaelis–Menten constant (*K*_m_) for an enzyme-substrate combination states the substrate concentration at half maximum reaction velocity for the enzyme-catalyzed conversion. A low *K*_m_ is an indication of an efficient substrate enzyme combination. The *K*_m_ for GSSG with GR is reported by the enzyme supplier as 0.06 mM [19] and our value of 0.07 mM is in good agreement with this. The reciprocal of the *K*_m_ constant can be regarded as an indication of the substrate affinity. The 1/*K*_m_ values for GR with GSSG and GSSA are 16.7 mM^−1^ and 2.0 mM^−1^, respectively, indicating that GSSG is approximately 8 × a better substrate for GR than is GSSA. Nevertheless, the reduction of GSSA by GR shows clear Michaelis–Menten kinetics and GSSA is thereby shown to be a substrate for GR.

### 3.3. A Δgsh1 Mutant Can Grow on Medium without GSH When Supplemented with GSSA

**Figure 3:**
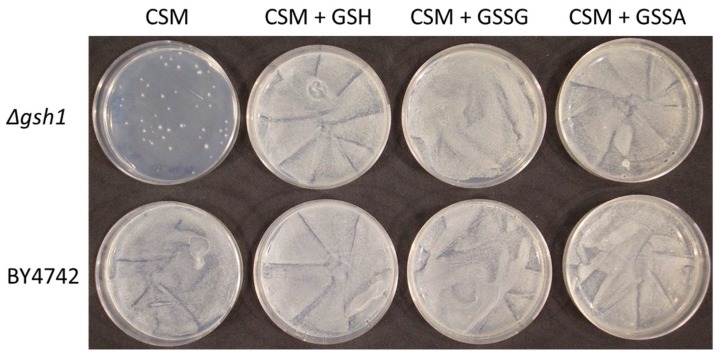
Growth of the wild type BY4742 yeast strain compared with the *Δgsh1* mutant on complete synthetic mixture (CSM) and CSM supplemented with 10 mM glutathione (GSH), 5 mM glutathione disulfide (GSSG) or 10 mM *S*-allylmercaptoglutathione (GSSA). A 100 µL aliquot of a logarithmically growing yeast culture adjusted to OD_600_ = 0.2 (OD = optical density) was spread onto the surface of each plate with a Drigalski spatula.

Mutants lacking γ-glutamylcysteine synthetase are auxotrophic for GSH and cannot grow without GSH-supplementation. GSSG can serve as a source of GSH because it can be reduced by the NADPH-dependent enzyme glutathione reductase (GR). In Figure 3 it can be seen that *S*-allylmercaptoglutathione (GSSA) can also serve as a source of GSH for the *Δgsh1* mutant, indicating that it is a substrate for GR in vivo. The individual yeast colonies growing on the CSM plate inoculated with the *Δgsh1* mutant are spontaneous mutations of *PRO2*, the gene coding for the second enzyme in the proline biosynthesis pathway, permitting the growth of *Δgsh1* in the absence of exogenous GSH [20]. It is interesting that, although GSSA is not as good a substrate for GR as GSSG, the *Δgsh1* mutant seems to grow equally well on both. This indicates that despite the different substrate affinities, sufficient GR activity is present in the yeast cells to provide adequate levels of GSH from GSSA for unrestricted growth, at least in absence of oxidative stress, as here. This is perhaps also an indication of the necessary efficiency of the cytosolic enzyme for its physiological function of reducing GSSG back to GSH with sufficient capacity to ensure that GSSG only builds up in those cell compartments where it is needed, e.g., in the oxidizing environment of the endoplasmic reticulum, where the reduction potential environment is more oxidized to facilitate cystine bridge formation, and in the vacuole where it can do no harm [3].

## 4. Conclusions

Cells exposed to sublethal doses of allicin, which targets the cellular glutathione pool, show a dose-dependent lag phase with subsequent growth recovery [17,21]. We conclude that it is probable that after exposure to sublethal doses of allicin, leading to conversion of part of the GSH pool to GSSA, reduction back to GSH by GR can be a component of the recovery of cell growth. The ability of GSSA to complement the GSH auxotrophy of the *Δgsh1* yeast mutant suggests that the reaction is physiologically relevant. Because garlic is a common foodstuff it would also be interesting to determine whether *S*-allylmercaptoglutathione is a substrate for human GR.

## Supplementary Materials

The following are available online at http://www.mdpi.com/2076-3921/7/7/86/s1, Figure S1: The kinetic measurements for the enzyme reactions of GR, Table S1: The final substrate concentration in the assay mix is given, the gradients were calculated from linear regions of the plots in Figure S1A, Table S2: The final substrate concentration in the assay mix is given, the gradients were calculated from linear regions of the plots in Figure S1B.
