# Supplementary material for: S-allylmercaptoglutathione Is a Substrate for Glutathione Reductase (E.C. 1.8.1.7) from Yeast (Saccharomyces cerevisiae)
Source: Antioxidants (Basel). 2018 Jul 6;7(7):86. doi: 10.3390/antiox7070086 (PMC6070820; doi:10.3390/antiox7070086)
Supplement: Supplementary file 1 [file antioxidants-07-00086-s001.pdf]

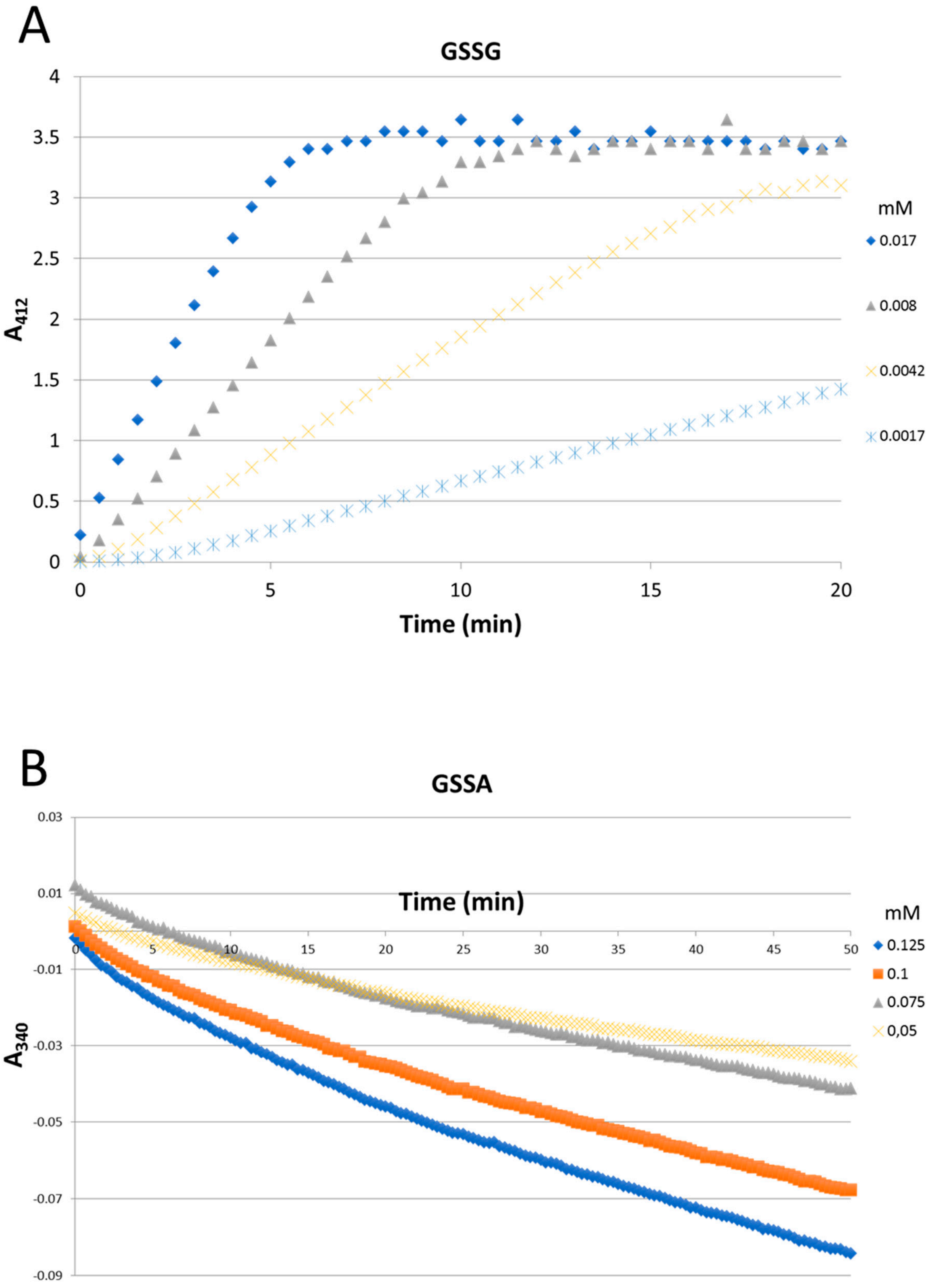

Figure S1. The kinetic measurements for the enzyme reactions of GR: **A**, with GSSG as substrate in the standard DTNB protocol (see M & M), and **B**, with GSSA as substrate without DTNB measuring NADPH consumption at A_{320}. The reagent blanks, in both cases without GR, were automatically subtracted from the test cuvette readings by the spectrophotomer program. The final substrate concentration in the reaction mix is indicated.

Table S1. The final substrate concentration in the assay mix is given. The gradients were calculated from linear regions of the plots in Figure S1A.

S [GSSG] mM	0.017	0.008	0.0042	0.0017
1/S	60	120	240	600
V = gradient	0.62	0.36	0.190	0.078
1/V	1.62	2.78	5.25	12.88

Note: 1/Km: 14.08; Km: 0.071.

Table S2. The final substrate concentration in the assay mix is given. The gradients were calculated from linear regions of the plots in Figure S1B.

S [GSSA] mM	0.125	0.10	0.75	0.50
1/S	8	10	13.3	20
V = gradient	−0.00132	−0.00112	−0.00083	−0.00060
1/V	758	894	1199	1662

Note: 1/Km: 2.00; Km: 0.50.
